# The association between spirituality and religiousness and mental health

**DOI:** 10.1038/s41598-018-35380-w

**Published:** 2018-11-22

**Authors:** Luciano Magalhães Vitorino, Giancarlo Lucchetti, Frederico Camelo Leão, Homero Vallada, Mario Fernando Prieto Peres

**Affiliations:** 1 School of Medicine, Federal University of Juiz de Fora, Juiz de Fora, Brazil; 2 Present Address: Faculty of Medicine of Itajubá, Itajubá, Minas Gerais, Brazil; 3 School of Medicine, Federal University of Juiz de Fora, Juiz de Fora, Brazil; 4 School of Medicine, University of São Paulo, São Paulo, Brazil

## Abstract

The present study aims to investigate how different levels of spirituality and religiousness (high spirituality and high religiousness –S/R, high spirituality and low religiousness –S/r, low spirituality and high religiousness s/R and low spirituality and low religiousness – s/r) are associated with quality of life, depressive symptoms, anxiety, optimism and happiness among adults. A cross-sectional study was carried out among 1,046 Brazilian adults. Concerning the different levels of spirituality and religiousness, 49.2% had s/r, 26.5% S/R, 17.2% S/r and 7.1% s/R. Participants with S/R had better outcomes as compared to those with s/r and those with S/r in WHOQOL Psychological, Social Relationship and Environment, Optimism and happiness. Participants with s/R had better outcomes as compared to those with s/r in WHOQOL Psychological and Social Relationship, Optimism and happiness. Participants with S/r were different from those with s/r, with higher levels of WHOQOL Environment and happiness but also anxiety. The results revealed that, having higher levels of both spirituality and religiousness were more correlated to better outcomes than having just one of them or none of them. Likewise, having higher levels of religiousness in detriment of higher levels of spirituality was also associated with better outcomes in comparison to others.

## Introduction

Several studies have been assessing the role of spirituality and religiousness (S/R) on well-being, quality of life (QoL), survival and physical and mental health worldwide^1–8^. Likewise, there is promising evidence that spirituality may enhance positive clinical outcomes in clinical practice^3,9–12^.

Despite the growing interest of the scientific community on S/R and health^13^, the literature still shows no consensus on the concepts of spirituality while there is a partial consensus for religiousness. Religiousness is defined as the “extension to which an individual believes, follows, and practices a religion^1^ and usually these beliefs influence how people seek to live out their lives and treat others^14^. Spirituality, on the other hand, is a more complex concept. According to Koenig *et al*.^1^, spirituality is to seek for a meaning in life, about the relations with the sacred or transcendent, and the connection with a higher power or supreme-being. A broader concept is provided by Puchalski *et al*.^15^ who defines spirituality as the aspect of humanity that refers to the way individuals seek and express meaning and purpose and the way they experience their connectedness to the moment, to self, to others, to nature, and to the significant or sacred. These different definitions have been discussed by several authors in the last decades and no consensus has been reached^15,16^.

Trying to better understand these concepts and whether they are important in the clinical practice, some studies started to investigate if there are differences between those with higher levels of religiousness but lower levels of spirituality and those with higher levels of spirituality and lower levels of religiousness^17^. However, the literature remains very controversial regarding this topic. On one hand, some authors have found that those with high levels of religiosity but low levels of spirituality had better health outcomes (i.e. low prevalence of substance and alcohol use, and lower levels of anxiety, phobia and others mental disorders)^18,19^. On the other hand, other authors have found the exact opposite, as those individuals with high levels of spirituality even with low levels of religiosity were associated with better health (i.e. better physical functioning, QoL, self-reported health status and less depressive symptoms)^20,21^.

Recently, a Brazilian study with 782 adults further investigated this area and used the Duke Religion Index to assess religiousness and the FACIT-SP 12 to assess spirituality. As a result, “meaning” and “peace” were more important and associated with QoL and mental health than religiousness levels. The participants with higher levels of religiousness, but lower levels of “meaning” and “peace” presented worse outcomes compared to those with lower levels of religiousness and higher levels of meaning or peace^2^. However, the FACIT-Sp 12 has been criticized by some authors due to overlapping concepts with well-being measures, and therefore it would be appropriate to investigate if self-report S/R measures would have the same results.

Understanding these differences could help to elucidate these concepts and the discussion in the field. However, despite the fact that thousands of studies have been published showing that spirituality and religiosity together could result in better outcomes, there are still few studies investigating which component, if any, would be more important. This could be relevant to the field of “Spirituality and Health” as well as to the clinical practice, since we will be able to understand individuals that carry out specific components of spirituality or religiosity, instead of including a “one size fits all” approach. Although these individuals are common in the clinical practice, they are seldom included in scientific studies and we have still little evidence concerning this matter. In addition, to our knowledge, there is a lack of studies trying to understand this concept of “spiritual but not religious” and “religious but not spiritual” in Latin America and within broader ethnic/religious contexts, given that most studies have involved American/European and Christian populations. Likewise, most published studies are including a more restricted concept of religiousness and spirituality. Finally, in the present study, the levels of S/R and mental health outcomes were assessed using a self-administered online survey, which could potentially minimize the social desirability bias of these participants, as noted in a previous study^22^.

Therefore, aiming to fill these gaps, the present study investigates how different levels of S/R (high spirituality and high religiousness – S/R, high spirituality and low religiousness – S/r, low spirituality and high religiousness s/R and low spirituality and low religiousness – s/r) are associated with QoL, depressive symptoms, anxiety, optimism and happiness amon^22^ g adults.

## Results

From 1,252 participants of this study, 1,046 (83.5%) completed all items of questionnaires. The mean age of participants was 40.7 years (SD 15.2). Table 1 shows the socio-demographic profile. Most participants were female (52%), 86.7% were between 18–59 years old and had a university level education (56.5%). Concerning the levels of spirituality and religiousness of each participant, they were allocated in four subgroups: 49.2% had s/r, 26.5% S/R, 17.2% S/r and 7.1% s/R. Table 2 presents the unadjusted mean scores of all outcomes.

**Table 1:** Participant’s socio-demographic characteristics (*N* = 1046).

Variables	n (%)
**Age group**	
18–24	228 (21.8)
25–39	309 (29.5)
40–59	370 (35.4)
≥60 years	139 (13.3)
**Gender**	
Female	544 (52.0)
Male	502 (48.0)
**Marital Status**	
Married	607 (58.0)
Widowhood/divorced	97 (9.3)
Single (never married)	342 (32.7)
**Education**	
With university graduate	591 (56.5)
No university graduate	455 (43.5)
**Spirituality and religiousness levels**	
Low spirituality and low religiousness	515 (49.2)
Low spirituality and high religiousness	74 (7.1)
High spirituality and low religiousness	180 (17.2)
High spirituality and high religiousness	277 (26.5)

**Table 2:** Mean^a^ scores for quality of life, depression, anxiety, optimism, happiness, religious beliefs, faith, meaning and peace (*N* = 1046).

Dependents Variables^a^	Mean (SD) CI 95%
**WHOQOL-BREF**	
Physical health	69.49 (17.31) 68.52–70.53
Psychological	67.20 (17.35) 66.19–68.24
Social relationships	64.68 (21.13) 63.44–66.01
Environment	60.43 (17.17) 59.43–61.49
**Depression (PHQ-9)**	
Total score	7.06 (6.02) 6.70–7.44
**Anxiety (GAD-7)**	
Total score	6.32 (5.29) 6.01–6.64
**Optimism – TOV-Revised**	
Total score	15.74 (2.38) 15.51–15.98
**Happiness**	
Happiness today	7.39 (2.09) 7.18–7.42
Happiness in future (5 years)	8.72 (1.57) 8.63–8.82
**P-Durel**	
OR	3.45 (1.58) 3.36–3.55
NOR	3.25 (1.70) 3.15–3.36
IR	6.68 (3.50) 6.49–6.90
Total score	13.40 (5.96) 13.03–13.75
**Facit**	
Faith	10.12 (4.26) 9.86–10.36
Meaning	11.55 (3.23) 11.36–11.75
Peace	10.44 (3.13) 10.26–10.63
Total score	32.12 (8.80) 31.59–32.66

^a^Unadjusted scores for all dependent variables. SD = Standard Deviation; CI 95%: Confidence Interval; WHOQOL = World Health Organization Quality of Life; PHQ-9 = Patient Health Questionnaire-9; GAD-7 = General Anxiety Disorder-7; LOT-Revised = Life Orientation Test-Revised; OR = Organizational religious; NOR = Nonorganizational religious; IR = Intrinsic religious.

In univariate GLM regression, Optimism - LOT-Revised (*F* = 5.42, *p* = 0.001) had a significant main effect, but Depression-PHQ-9 (*F* = 1.06, *p* = 0.535) and Anxiety-GAD-7 (*F* = 1.59, *p* = 0.189) had not. Multivariate GLM regression model revealed a significant main effect for the domains of WHOQOL-BREF with Wilks’s Lambda = 0.953, *F* = 4.221, *p* < 0.001; dimensions of Happiness (Wilks’s Lambda = 0.954, *F* = 8.308, *p* < 0.001); subscales of DUREL (Wilks’s Lambda = 0.736, *F* = 37.635, *p* < 0.001) and sub-domains of FACIT (Wilks’s Lambda = 0.803, *F* = 26.429, *p* < 0.001). Covariate-adjusted mean score for all dependent variables and mean differences scores for levels of S/R are reported in Table 3.

**Table 3:** Effect of different levels of spirituality and religiousness on quality of life and mental health aspects (*N* = 1046).

Dependent Variables	Mean (SD)				*F*	*p*	Mean differences
WHOQOL-BREF	Sr	sR	Sr	SR			
Physical health	68.85 (16.91)	70.18 (18.60)	69.22 (18.20)	70.68 (18.81)	1.01	0.385	sr x sR p = 0.460; sr x Sr p = 0.767; sr × RS p = 0.093
							Rs × rS p = 0.631; Rs × RS p = 0.792; rS × RS p = 0.295
Psychological	64.94 (17.43)	68.81 (18.45)	66.78 (17.46)	71.26 (15.23)	11.972	**<0.001**	sr × sR **p = 0.029**; sr × Sr p = 0.135; sr × SR **p < 0.001**
							SR × Sr p = 0.301; sR × SR p = 0.190; Sr × SR p = 0.001
Social relationships	61.91 (20.74)	68.57 (22.01)	64.08 (21.65)	69.18 (19.69)	9.657	**<0.001**	sr × sR **p = 0.005**; sr × Sr p = 0.188; sr × SR **p < 0.001**
							sR × Sr p = 0.089; sR × SR p = 0.808; Sr × SR **p = 0.006**
Environment	58.52 (16.54)	60.34 (18.66)	60.60 (17.16)	63.89 (16.82)	7.916	**<0.001**	sr × sR p = 0.317; sr × Sr **p = 0.002**; sr × SR **p < 0.001**
**Depression (PHQ-9)**							sR × Sr p = 0.899; sR × SR p = 0.065; Sr × SR **p = 0.020**
Total score	7.01 (5.98)	6.57 (5.85)	7.55 (6.27)	6.97 (5.94)	0.728	0.535	sr × sR p = 0.515; sr × Sr p = 0.250; sr × SR p = 931
**Anxiety (GAD-7)**							sR × Sr p = 0.191; sR × SR p = 0.571; Sr × SR p = 0.270
Total score	6.09 (5.28)	6.66 (5.34)	6.97 (5.58)	6.25 (5.08)	1.596	0.189	sr × sR p = 0.351; sr × Sr **p = 0.037**; sr × SR p = 0.678
**Optimism - LOT-Revised**							sR × Sr p = 0.636; sR × SR p = 0.519; Sr × SR p = 0.120
Total score	15.35 (3.85)	16.33 (3.53)	15.62 (3.98)	16.40 (3.81)	5.423	**0.001**	sr × sR **p = 0.033**; sr × Sr p = 0.411; sr × SR **p < 0.001**
**Happiness**							sR × Sr p = 0.160; sR × SR p = 0.819; Sr × SR **p = 0.028**
Today	7.08 (2.07)	7.58 (2.42)	7.18 (2.37)	7.75 (1.61)	1.307	**0.002**	sr × sR **p = 0.029**; sr × Sr **p = 0.036**; sr × SR **p < 0.001**
							sR × Sr p = 0.063; sR × SR p = 0.486; Sr × SR p < 0.001
In future (5 years)	8.44 (1.72)	8.95 (1.50)	8.79 (1.62)	9.13 (1.03)	1.293	**0.002**	sr × sR **p = 0.006**; sr × Sr **p = 0.007**; sr × SR **p < 0.001**
**PDurel**							sR × Sr p = 0.433; sR × SR p = 0.341; Sr × SR **p = 0.016**
OR	3.97 (1.50)	2.73 (1.41)	3.67 (1.47)	2.53 (1.31)	67.223	**<0.001**	sr × sR **p < 0.001**; sr × Sr **p = 0.017**; sr × SR **p < 0.001**
							sR × Sr **p < 0.001**; sR × SR p = 0.285; Sr × SR **p < 0.001**
NOR	3.87 (1.71)	2.60 (1.30)	3.18 (1.67)	2.30 (1.14)	67.100	**<0.001**	sr × sR **p < 0.001**; sr × Sr **p < 0.001**; sr × SR **p < 0.001**
							sR × Sr **p = 0.006**; sR × SR **p = 0.136**; Sr × SR **p < 0.001**
IR	8.17 (3.83)	4.72 (1.49)	6.69 (2.74)	4.45 (1.44)	1003.9	**<0.001**	sr × sR **p < 0.001**; sr × Sr **p < 0.001**; sr × SR **p < 0.001**
							sR × Sr **p < 0.001**; sR × SR p = 0.476; Sr × SR **p < 0.001**
**Total score**	**16.16 (6.24)**	**9.94 (3.14)**	**13.60 (4.73)**	**9.03 (2.87)**	**4.24**	**0.04**	**sr × sR p < 0.001; sr × Sr p < 0.001; sr × SR p < 0.001**
**Facit**							**sR × Sr p < 0.001; sR × SR p = 0.996; Sr × SR p < 0.001**
Meaning	10.92 (3.32)	11.70 (2.97)	11.80 (3.09)	12.52 (2.80)	18.352	**<0.001**	sr × sR **p = 0.031**; sr × Sr **p = 0.001**; sr × SR **p < 0.001**
							sR × Sr **p < 0.807**; sR × SR **p = 0.033**; Sr × SR **p = 0.011**
Peace	9.97 (3.15)	10.43 (3.16)	10.67 (3.08)	11.18 (2.85)	11.527	**<0.001**	sr × sR **p = 0.190**; sr × Sr **p = 0.004**; sr × SR **p < 0.001**
							sR × Sr **p = 526**; sR × SR **p = 0.039**; Sr × SR **p = 0.057**
Faith	8.40 (4.35)	12.26 (2.94)	10.57 (3.65)	12.44 (2.85)	82.202	**<0.001**	sr × sR **p < 0.001**; sr × Sr **p < 0.001**; sr × SR **p < 0.001**
							sR × Sr **p = 0.001**; sR × SR **p = 0.699**; Sr × SR **p < 0.001**
**Total escore**	**28.93 (8.71)**	**34.55 (7.91)**	**32.82 (7.88)**	**36.95 (7.15)**	**91.667**	**<0.001**	**sr × sR p < 0.031; sr × Sr p = 0.001; sr × SR p < 0.001**
							**sR × Sr p = 0.807; sR × SR p = 0.033; Sr × SR p = 0.011**

Note: SD = Standard deviation; S/r = high spirituality and low religiousness level; s/R = low spirituality and high religiousness level; S/R = high spirituality and high religiousness level and r/s = low spirituality and low religiousness level. Covariates: age, gender, marital status, education and perceived health.

Note: SD = Standard deviation; S/r = high spirituality and low religiousness level; s/R = low spirituality and high religiousness level; S/R = high spirituality and high religiousness level and r/s = low spirituality and low religiousness level. Covariates: age, gender, marital status, education and perceived health.

Participants with high spirituality and high religiousness (S/R) had better outcomes as compared to those with low spirituality and low religiousness (s/r) and compared to those with high spirituality and low religiousness (S/r) in the following instruments: WHOQOL Psychological, Social Relationship and Environment, Optimism and happiness. Participants with low spirituality and high religiousness (s/R) had better outcomes as compared to those with s/r in the following instruments: WHOQOL Psychological and Social Relationship, Optimism and happiness. Participants with S/r were different from those with s/r, with higher levels of anxiety, WHOQOL Environment and happiness. Finally, we detected no differences between those with s/R compared to those with S/r and between those with s/R as compared to those with S/R.

## Discussion

The present study has investigated whether different levels of spirituality and religiousness were associated with several health outcomes. Our results revealed that, in general, having higher levels of both spirituality and religiousness were associated with better outcomes than having just one of them or none of them. Likewise, having higher levels of religiousness in detriment of higher levels of spirituality were also associated with better outcomes in comparison to other levels. Higher levels of spirituality but not religiousness were associated with better social and environmental QoL, but also with more anxiety. Finally, lower levels of both spirituality and religiousness were associated with worse outcomes. These results advance further the understanding of the concepts of spirituality and religiousness and will be explored below.

The fact that those with S/R have been associated with higher QoL, optimism and happiness when compared to people with s/r level is widely supported by the current scientific literature^2–4,23,24^. The practice of an organized religion (e. g., public or private religious practice) or individual religion, formally structured, with doctrines to be followed by common group, enhance social support, healthy behaviors, better lifestyle and happiness. Likewise, religiousness and spirituality also help to deal with stress situations, fear, anguish, sadness, fury, and anger^1,25^. It is believed that individuals with high S/R levels tend to have better mental health and QoL outcomes because they develop internal and external mechanisms that help them cope with the adversities on life course^1,24,25^. Spirituality and religiousness are also components of people’s psychological well-being and there is evidence showing an association between low levels of spiritual and religious beliefs with mental health impairment^26^.

In fact, there is strong evidence that S/R together generally leads to better outcomes. However, if these positive outcomes would persist even after a missing component is still under investigation. In our study, the study population with s/R level, although with lower intensity than those with S/R level, presented better self-perception of QoL, optimism and happiness when compared against the study population with S/r and s/r levels, suggesting that higher levels of religious practices seems to be more important for QoL and mental health aspects than the levels of spirituality. A possible explanation for these findings is that group activities, coexistence with other people with the same belief, and philosophy of life can unleash functional behaviors, positive feelings, social support and emotion of gratitude^1,26,27^.

Comparing these results with previous studies, we found that this is a very controversial topic in the scientific community. A previous Brazilian study including 782 adults showed that “meaning” and “peace” (components of the FACIT-Sp 12 spiritual well-being scale) were more strongly associated with health outcomes, in a sense that those with high levels of intrinsic religiosity but low levels of meaning/peace have worse outcomes than those with low religiousness and high meaning/peace. The authors also found that religious participants found greater meaning and peace than nonreligious participants^2^. Therefore, the previous study concluded that the components of spirituality (meaning and peace) were more important than faith and religiousness. These findings are different from ours and we believe that the most probable reason is that we used a self-reported spirituality measure instead of a spiritual well-being scale. This approach was designed to avoid the tautological criticisms of this kind of questionnaire (i.e. using some questions associated with well-being).

Other international studies have also investigated this topic with conflicting results. A British study conducted between 2006 and 2007, investigated the association between spirituality and religiosity and psychiatric symptoms among 7,403 persons. Authors found that those participants with higher spirituality and lower religiousness were more prone to suffer from mental disorders, such as generalized anxiety disorder (OR = 1.50, 95% CI 1.09–2.06), phobia (OR = 1.72, 95% CI 1.07–2.77) and neurotic disorders (OR = 1.37, 95% CI 1.12–1.68)^18^. Same results were noted by Leurent *et al*.^19^ who evaluated 8,318 people from seven countries and found that people who were spiritual but not religious were 2.73 times more likely to develop depression than those who were neither religious nor spiritual. In the same direction, a recent American study including 1,013 healthy adults identified that people who self-rated spiritual but not religious were more likely to hold paranormal and supernatural beliefs and had greater schizophrenia symptoms than religious or non-religious people^28^.

On the other hand, some authors have found the opposite. Another British study investigated 203 participants and found that people who had higher levels of spirituality showed significantly stronger social support and did not present depressive symptoms and anxiety when compared with religious persons. Likewise, an American study carried out in 277 older adults identified that geriatric outpatients who report greater spirituality (OR = 1.15, 95% CI 1.10–1.21, P < 0.01), but not religiosity (OR = 0.93, 95% CI 0.85–1.02, P = 0.12), were more likely to perceive their health as good^20^.

Although we found no consensus in this area of research as reported above, we have some hypotheses to our findings. First, people who have greater prevalence of spiritual experiences (e. g. have mystical or supernatural experiences and feelings of universal connectedness), but do not profess an organized religion may have a certain predisposition to mental disorders^29^, such as, Schizophrenia^28,30–32^ and depression^19,33,34^. Second, according to Saucier and Skrzypińska^35^ people with greater spirituality but with lower levels of religiousness are more prone to present fantasy-proneness, dissociation, and beliefs of a magical or superstitious sort, as well as eccentricity and high openness to mystical experiences. This kind of people can have difficulty to follow an organized religions practices, because it is necessary to follow traditionalism and, more moderately, with collectivism versus individualism and with low openness to experience.

Our study has some limitations that should be explored. This is a cross-sectional study, avoiding cause-effect conclusions. A longitudinal approach would be important to understand if those participants with high spirituality but low religiosity are those who do not fit in organized traditions due to mental health problems. Another limitation of our study is the fact that data collection was carried out only in people who had online access to email, social media or online websites. Nevertheless, when comparing our study with other studies that have investigated the general population, our findings revealed similar scores concerning the levels of QoL^36,37^, depression^38^, anxiety^39,40^, optimism^41^, religiousness^42^ and faith, peace and meaning^43^, which supports the generability of our data. Despite these limitations, this study has addressed a significant gap in understanding the associations of different levels of spirituality and religiousness of adults’ QoL and mental health.

In conclusion, our findings revealed that having high levels of both spirituality and religiosity are associated with better QoL (psychological, social and environment), optimism, and happiness as compared to those having only spirituality, only religiousness or none of them. Nevertheless, it seems that having high levels of religiousness instead of high levels of spirituality is more related to better outcomes. These findings could bring the modern medicine to the World Health Organization’s definition of health as “a state of complete physical, mental and social well-being and not merely the absence of disease or infirmity”^44^. In addition, the finds may help in the future discussion of the definitions and concepts in the field.

## Methods

### Study Design

A cross-sectional online study was undertaken between June and August 2016 and is part of the project “Spiritual and Religious Beliefs, Practices and Experiences in the General Population” sponsored by “Brazilian Interfaith Coalition on Spirituality and Health” (coalizaointerfe.org). This non-profit coalition is composed of health care professionals and representative members of religious or non-religious faith practices. This study was approved by the Institutional Review Board of the Albert Einstein Hospital (São Paulo, Brazil) and all participants gave online informed consent. All study procedures involving humans were performed in accordance with the ethical regulations of the institutional and Brazilian national research committee and with the 1964 Helsinki declaration.

### Study population, sample and procedures

Qualtrics® (https://www.qualtrics.com) sent invitations to participate in the survey through its panel partner organizations to the targeted Brazilian population, inviting the study population to complete an online survey. As surveys were being completed, response patterns were monitored and decisions were made about sampling in order to meet the establish population quota. Quotas were set in order to limit the respondents according to social class distribution, age, gender and geographic location so the population surveyed could meet the same profile of the general adult population in Brazil, according to the 2010 census^45^.

Quality check questions and attention filters were added. Questions were divided into five randomized blocks so the impact of tiredness of respondents equally affected all questions. Forced response validation was included in all questions. The length of interview was less than 30 minutes. The study population included if they had online access to email, social medias or online websites. Participants were excluded from the study if they have missing data, if their email was not valid, if they did not reply or sent incomplete answers to the questionnaire.

### Independent variables

The questionnaire included the following instruments:

- A sociodemographic questionnaire: including age (years), gender (female and male), marital status (married; widowhood/divorced; single) and education level (with university graduation and no university graduation).
- Self-reported levels of spirituality and religiousness: In order to investigate the levels of S/R, two questions were used: “How religious are you?” and “How spiritual are you?“ with the possible answers: “1 (not at all), 2 (a little bit), 3 (moderately), 4 (quite a lot), and 5 (very much)”. For the present study, we separated the levels of S/R as follows^46,47^: high spirituality and low religiousness (S/r) – those who answered “quite a lot” or “very much” for spirituality and “not at all”, “a little bit” and “moderately” for religiousness; low spirituality and high religiousness (s/R) – those who answered “quite a lot” or “very much” for religiousness and “not at all”, “a little bit” and “moderately” for spirituality; high spirituality and high religiousness (S/R) – those who answered “quite a lot” or “very much” for spirituality and religiousness; and low spirituality and low religiousness (s/r) – those who answered “not at all”, “a little bit” and “moderately” for spirituality and religiousness.
- Self-reported health status: The self-reported health status was measured using a single question from the Brazilian version of the WHOQOL-BREF^48^. The question was Q2 = “How satisfied are you with your health?” (1 = very dissatisfied; 2 = dissatisfied; 3 = neither satisfied nor dissatisfied; 4 = satisfied; 5 = very satisfied). Item response reflects assessments over the past 2 weeks, on a 5-point Likert-type scale. The higher the Likert Scale score, means the higher self-perceived health^48,49^.

- QOL was assessed using the Brazilian version of the WHOQOL-BREF^48,49^. This generic instrument of QOL with 26-item (5-point Likert), two of which are general and the others representing each one of the 24 aspects which make up the original instrument, which covers four domains: physical, psychological, social relationships and the environment. The higher score represents better self-perception of QOL; however, there is no cut-off point for its classification. The Brazilian version presented good reliability (α = 0.77)^48^. In the present study, the Cronbach’s alpha revealed high level (α = 0.85).
- Depression was screened using the “Patient Health Questionnaire-9” (PHQ-9)^50^, this instrument was validated in Brazil^51^. PHQ-9 has 9-item to evaluate the frequency of depressive symptoms in the last two weeks using the DSM-IV criteria as “0” (not at all) to “3” (nearly every day). PHQ-9 presented excellent reliability in previous Brazilian studies (α ≥ 0.80)^52,53^.
- Anxiety was assessed through “General Anxiety Disorder (GAD-7)”^54^ validated for the Brazilian population^55^. This is a 7-item instrument investigating anxiety disorders over the past two weeks using the DSM-IV criteria as “0” (not at all) to “3” (nearly every day). GAD-7 presents excellent reliability among Brazilian adults (α = 0.916)^56^.
- Optimism using the Life Orientation Test-Revised (LOT-R)^41^, validated for the Brazilian population^57^. This instrument evaluates individual differences in optimism/pessimism. LOT-R has 10-item, consisting of three items for optimism, three for pessimism and four filler items. Each item is rated on a 5-point Likert scale from strongly agree to strongly disagree. The total sum score was calculated by adding the raw scores of the optimism subscale with the inverted pessimism raw scores^58^. LOT-R presents appropriate reliability (Optimism α = 0.70 and Pessimism α = 0.80)^59^. In the present study the LOT-R total scale Cronbach’s alpha coefficients was α = 0.80.
- Happiness was assessed through the “Subjective Happiness Scale”^60^ validated into Portuguese^61^. This scale has a 4-item (7-point likert) scale, measuring the subjective happiness. This scale presented reasonable reliability in the Brazilian validation (α = 0.67)^61^. In this study the Cronbach’s alpha showed slightly higher (α = 0.70).
- Religiousness was assessed through the Duke University Religion (DUREL) Index^62^ validated into Portuguese and called PDUREL^42^. This is a 5-item measure of religious involvement, which capture three subscales: (1) Organizational religious behavior (public religious activities) (1 item), (2) Nonorganizational religious behavior (religious activities performed in private, such as prayer) (1 item) and (3) Intrinsic religious motivation (pursuing religion as an ultimate end in itself). Higher scores represent lower levels of religiousness. The Brazilian version and the present study showed a good internal consistency: α = 0.73^42^ and α = 0.75 respectively.
- Faith, peace and meaning were assessed through the FACIT-Sp 12^63^ validated for Brazilian population^64^. It is a self-administered questionnaire composed of 12 items, divided equally between three dimensions: Meaning, Peace and Faith. Participants assess the items on a five-point Likert scale (from 0 “not at all” to 4 “enormously”). The questionnaire provides four scores: one per dimension and one global. A high global score reflects a higher level of faith, peace and meaning. Participants were instructed to indicate how true an item had been for them during the past 7 days, using a 5-item response format ranging from not at all (0) to very much (4). Some examples of statements include: “I feel peaceful” (Peace), “I have a reason to Live” (Meaning) and “I find comfort in my faith or spiritual beliefs” (Faith). FACIT-Sp 12 demonstrated high reliability in Brazilian context (α = 0.89)^64^ and good reliability in this study (α = 0.75).

### Data analysis

Data was analyzed using the Statistical Package for Social Sciences - SPSS 23 (SPSS Inc.). We performed the descriptive statistics for the socio-demographic characteristics, levels of spirituality and religiousness, health and for all dependents variables (QoL, depression, anxiety, optimism, happiness, religious beliefs, faith, meaning and peace).

First, an univariate General Linear Models (GLM)^65^ was used to compare the scores of depression (PHQ-9 total score), anxiety (GAD-7 total score) and Optimism (LOT-Revised total score), through the four different levels of spirituality and religiousness (S/r; s/R; S/R and s/r). Then, a multivariate GLM, procedure used to assess multi-level effects was used for the following dependent variables: QoL (four domains of WHOQOL-BREF), happiness (two items of Subjective Happiness Scale), religiousness (three subscales of PDUREL) and Faith, Peace and Meaning (three sub-domains of FACIT-Sp 12) in order to compare the same four levels of spirituality and religiousness. These levels of spirituality and religiousness were treated as fixed factors and the models were adjusted for the following covariates: age, gender, marital status, education and self-reported health status, resulting in covariate-adjusted mean scores for the dependents variables. Data was also evaluated for linearity, multicollinearity, homogeneity of variance–covariance matrices, and outliers^66^. A significance level of 5% was chosen for the test, with a 95% confidence interval.

## References

[1] Koenig, H. G., King, D. & Carson, V. B. *Handbook of religion and health*. (Oup Usa, 2012).

[2] Peres, M. F. P., Kamei, H. H., Tobo, P. R. & Lucchetti, G. Mechanisms Behind Religiosity and Spirituality’s Effect on Mental Health, Quality of Life and Well-Being. *Journal of religion and health*, 1–14 (2017). 10.1007/s10943-017-0400-6 28444608

[3] de Bernardin Gonçalves JP, Lucchetti G, Menezes PR, Vallada H (2017). Complementary religious and spiritual interventions in physical health and quality of life: A systematic review of randomized controlled clinical trials. PloS one.

[4] Panzini RG (2017). Quality-of-life and spirituality. International Review of Psychiatry.

[5] Li S, Stampfer MJ, Williams DR, VanderWeele TJ (2016). Association of religious service attendance with mortality among women. JAMA internal medicine.

[6] Idler E, Blevins J, Kiser M, Hogue C (2017). Religion, a social determinant of mortality? A 10-year follow-up of the Health and Retirement Study. PloS one.

[7] Das S, Punnoose VP, Doval N, Nair VY (2018). Spirituality, religiousness and coping in patients with schizophrenia: A cross sectional study in a tertiary care hospital. Psychiatry research.

[8] Vitorino LM, Low G, Vianna LAC (2016). Linking Spiritual and Religious Coping With the Quality of Life of Community-Dwelling Older Adults and Nursing Home Residents. Gerontology & geriatric medicine.

[9] Koenig Harold G. (2012). Religion, Spirituality, and Health: The Research and Clinical Implications. ISRN Psychiatry.

[10] Lucchetti G, Bassi RM, Lucchetti ALG (2013). Taking spiritual history in clinical practice: a systematic review of instruments. Explore: The Journal of Science and Healing.

[11] Moreira-Almeida A, Koenig HG, Lucchetti G (2014). Clinical implications of spirituality to mental health: review of evidence and practical guidelines. Revista Brasileira de Psiquiatria.

[12] Oxhandler, H. K. & Parrish, D. E. Integrating clients’ religion/spirituality in clinical practice: A comparison among social workers, psychologists, counselors, marriage and family therapists, and nurses. *Journal of clinical psychology* (2017). 10.1002/jclp.22539 29023713

[13] Lucchetti G, Lucchetti ALG (2014). Spirituality, religion, and health: Over the last 15 years of field research (1999–2013). The International Journal of Psychiatry in Medicine.

[14] Parker M (2003). Religiosity and mental health in southern, community-dwelling older adults. Aging & Mental Health.

[15] Puchalski C (2009). Improving the quality of spiritual care as a dimension of palliative care: the report of the Consensus Conference. Journal of palliative medicine.

[16] King MB, Koenig HG (2009). Conceptualising spirituality for medical research and health service provision. BMC Health Services Research.

[17] Lucchetti G, Koenig HG, Pinsky I, Laranjeira R, Vallada H (2015). Spirituality or religiosity: is there any difference?. Revista Brasileira de Psiquiatria.

[18] King M (2013). Religion, spirituality and mental health: results from a national study of English households. The British Journal of Psychiatry.

[19] Leurent B (2013). Spiritual and religious beliefs as risk factors for the onset of major depression: an international cohort study. Psychological medicine.

[20] Daaleman TP, Perera S, Studenski SA (2004). Religion, spirituality, and health status in geriatric outpatients. The Annals of Family Medicine.

[21] Farias M, Underwood R, Claridge G (2013). Unusual but sound minds: mental health indicators in spiritual individuals. British Journal of Psychology.

[22] Peres MFP (2018). Religious landscape in Brazil: Comparing different representative nationwide approaches to obtain sensitive information in healthcare research. SSM - population health.

[23] Anderson JW, Nunnelley PA (2016). Private prayer associations with depression, anxiety and other health conditions: an analytical review of clinical studies. Postgraduate medicine.

[24] McFarland MJ (2009). Religion and mental health among older adults: Do the effects of religious involvement vary by gender?. Journals of Gerontology Series B: Psychological Sciences and Social Sciences.

[25] Pargament K, Feuille M, Burdzy D (2011). The Brief RCOPE: Current psychometric status of a short measure of religious coping. Religions.

[26] Weber Samuel, Lomax James, Pargament Kenneth (2017). Healthcare Engagement as a Potential Source of Psychological Distress among People without Religious Beliefs: A Systematic Review. Healthcare.

[27] Agli O, Bailly N, Ferrand C (2015). Spirituality and religion in older adults with dementia: a systematic review. International Psychogeriatrics.

[28] Willard AK, Norenzayan A (2017). “Spiritual but not religious”: Cognition, schizotypy, and conversion in alternative beliefs. Cognition.

[29] Baetz M, Bowen R, Jones G, Koru-Sengul T (2006). How spiritual values and worship attendance relate to psychiatric disorders in the Canadian population. The Canadian Journal of Psychiatry.

[30] Hergovich A, Schott R, Arendasy M (2008). On the relationship between paranormal belief and schizotypy among adolescents. Personality and Individual Differences.

[31] Schofield K, Claridge G (2007). Paranormal experiences and mental health: Schizotypy as an underlying factor. Personality and Individual Differences.

[32] Swami V, Pietschnig J, Stieger S, Voracek M (2011). Alien psychology: Associations between extraterrestrial beliefs and paranormal ideation, superstitious beliefs, schizotypy, and the Big Five personality factors. Applied Cognitive Psychology.

[33] Haddad M, Waqas A, Qayyum W, Shams M, Malik S (2016). The attitudes and beliefs of Pakistani medical practitioners about depression: a cross-sectional study in Lahore using the Revised Depression Attitude Questionnaire (R-DAQ). BMC psychiatry.

[34] Park J-I, Hong JP, Park S, Cho M-J (2012). The relationship between religion and mental disorders in a Korean population. Psychiatry investigation.

[35] Saucier G, Skrzypińska K (2006). Spiritual but not religious? Evidence for two independent dispositions. Journal of Personality.

[36] Purba FD (2018). Quality of life of the Indonesian general population: Test-retest reliability and population norms of the EQ-5D-5L and WHOQOL-BREF. PLoS One.

[37] Cruz LN, Polanczyk CA, Camey SA, Hoffmann JF, Fleck MP (2011). Quality of life in Brazil: normative values for the WHOQOL-bref in a southern general population sample. Quality of life research: an international journal of quality of life aspects of treatment, care and rehabilitation.

[38] Muñoz-Navarro, R. *et al*. Utility of the PHQ-9 to identify major depressive disorder in adult patients in Spanish primary care centres. *BMC Psychiatry* **17**, 10.1186/s12888-017-1450-8 (2017). 10.1186/s12888-017-1450-8 PMC5550940 28793892

[39] Jordan P, Shedden-Mora MC, Lowe B (2017). Psychometric analysis of the Generalized Anxiety Disorder scale (GAD-7) in primary care using modern item response theory. PLoS One.

[40] Zhong QY (2015). Diagnostic Validity of the Generalized Anxiety Disorder - 7 (GAD-7) among Pregnant Women. PLoS One.

[41] Scheier MF, Carver CS, Bridges MW (1994). Distinguishing optimism from neuroticism (and trait anxiety, self-mastery, and self-esteem): a reevaluation of the Life Orientation Test. Journal of personality and social psychology.

[42] Lucchetti G (2012). Validation of the duke religion index: DUREL (Portuguese version). Journal of religion and health.

[43] Peterman AH (2014). Measuring meaning and peace with the FACIT-spiritual well-being scale: distinction without a difference?. Psychological assessment.

[44] Organization WH (2017). Constitution of WHO: principles. About WHO. Viitattu.

[45] Demográfico IC (2010). Instituto Brasileiro de Geografia e Estatística. Acesso em.

[46] Curlin FA, Lantos JD, Roach CJ, Sellergren SA, Chin MH (2005). Religious characteristics of U.S. physicians: a national survey. J Gen Intern Med.

[47] Kørup AK (2017). The International NERSH Data Pool—A methodological description of a data pool of religious and spiritual values of health professionals from six continents. Religions.

[48] Fleck M (2000). Application of the Portuguese version of the abbreviated instrument of quality life WHOQOL-bref. Revista de saude publica.

[49] Group W (1998). Development of the World Health Organization WHOQOL-BREF quality of life assessment. Psychological medicine.

[50] Kroenke K, Spitzer RL, Williams JB (2001). The phq‐9. Journal of general internal medicine.

[51] Santos IS (2013). Sensitivity and specificity of the Patient Health Questionnaire-9 (PHQ-9) among adults from the general population. Cadernos de Saúde Pública.

[52] Camara RA, Kohler CA, Frey BN, Hyphantis TN, Carvalho AF (2017). Validation of the Brazilian Portuguese version of the Premenstrual Symptoms Screening Tool (PSST) and association of PSST scores with health-related quality of life. Revista brasileira de psiquiatria (Sao Paulo, Brazil: 1999).

[53] Chagas MH (2013). Validation and internal consistency of Patient Health Questionnaire-9 for major depression in Parkinson’s disease. Age and ageing.

[54] Spitzer RL, Kroenke K, Williams JB, Löwe B (2006). A brief measure for assessing generalized anxiety disorder: the GAD-7. Archives of internal medicine.

[55] Bergerot CD, Laros JA, Araujo TCCFd (2014). Assessment of anxiety and depression in cancer patients: a psychometric comparison. Psico-USF.

[56] Moreno André Luiz, DeSousa Diogo A., Souza Ana Maria Frota Lisbôa P., Manfro Gisele G., Salum Giovanni A., Koller Silvia H., Osório Flávia L., Crippa José Alexandra S. (2016). Factor Structure, Reliability, and Item Parameters of the Brazilian-Portuguese Version of the GAD-7 Questionnaire. Temas em Psicologia.

[57] Bandeira Marina, Bekou Valentin, Lott Keli Silva, Teixeira Marcela Augusta, Rocha Sandra Silva (2002). Validação transcultural do teste de orientação da vida (TOV-R). Estudos de Psicologia (Natal).

[58] Zenger M (2013). Evaluation of the Latin American version of the Life orientation Test-Revised. International Journal of Clinical and Health Psychology.

[59] Ottati F, Noronha APP (2017). Factor structure of the Life Orientation Test-Revised (LOT-R). Acta Colombiana de Psicología.

[60] Lyubomirsky S, Lepper HS (1999). A measure of subjective happiness: Preliminary reliability and construct validation. Social indicators research.

[61] Rodrigues A, Silva JAd (2010). The role of sociodemographic characteristics on happiness. Psico-USF.

[62] Koenig, H., Parkerson, G. R. Jr. & Meador, K. G. Religion index for psychiatric research. *The American journal of psychiatry* **154**, 885–886 (1997). 10.1176/ajp.154.6.885b 9167530

[63] Peterman AH, Fitchett G, Brady MJ, Hernandez L, Cella D (2002). Measuring spiritual well-being in people with cancer: the functional assessment of chronic illness therapy—Spiritual Well-being Scale (FACIT-Sp). Annals of behavioral medicine.

[64] Lucchetti G, Lucchetti ALG, de Bernardin Gonçalves JP, Vallada HP (2015). Validation of the Portuguese Version of the Functional Assessment of Chronic Illness Therapy–Spiritual Well-Being Scale (FACIT-Sp 12) Among Brazilian Psychiatric Inpatients. Journal of religion and health.

[65] Taylor, A. Using the GLM procedure in SPSS. *Macquarie: Macquarie University* (2011).

[66] Tabachnick, B. G. & Fidell, L. S. Computer-assisted research design and analysis. Vol. 748 (Allyn and Bacon Boston, 2001).

